# Ecological network collapse and functional potential shifts in the canine oral microbiota associated with periodontal disease: a pilot study

**DOI:** 10.1038/s41598-026-46400-5

**Published:** 2026-04-01

**Authors:** Jeongwoong Park, Seon-Ae Choi, Dohyun Kim, Jung-Hwan Ji, GuiHwan Han, Donghyun Shin, Seonghun Im

**Affiliations:** 1 Department of Animal Biotechnology, Jeonbuk National University, Jeonju, 54896 Republic of Korea; 2 Center for Industrialization of Agricultural and Livestock Microorganisms (CIALM), Jeongeup-si, Republic of Korea; 3 Department of Agricultural Convergence Technology, Jeonbuk National University, Jeonju, 54896 Republic of Korea; 4 The Korean Society of Animal Big Data Research, Korean Society of Animal Science and Technology, Seoul, 06367 Republic of Korea

**Keywords:** 16S rRNA gene sequencing, Alpha diversity, Canine oral microbiota, Dysbiosis, Ecological network, Periodontal disease, Computational biology and bioinformatics, Diseases, Microbiology

## Abstract

Periodontal disease is a highly prevalent disease in dogs; however, the underlying shifts in the oral microbiota remain unclear. Although dysbiosis is often associated with a loss of diversity, the ecological dynamics of canine periodontal disease remain poorly characterized. Therefore, we performed pilot study using a multi-angle comparison of the oral microbiota of healthy dogs and those with periodontal disease to identify the taxonomic, predicted functional, and structural signatures of the disease. Supragingival plaque samples were collected from five clinically healthy dogs and five dogs with periodontal disease. Microbial composition was analyzed by sequencing the 16S rRNA gene. Downstream bioinformatics analyses included alpha and beta diversity, differential abundance testing (DESeq2), functional prediction (Phylogenetic Investigation of Communities by Reconstruction of Unobserved States 2), and co-occurrence network analysis. Our results showed that the oral microbiota of the two groups were structurally distinct (permutational multivariate analysis of variance, *p* < 0.01), even after adjusting for age. Unexpectedly, the periodontal disease group exhibited significantly higher alpha diversity than the healthy group (*p* < 0.05). A high abundance of *Pasteurellaceae* characterized the healthy microbiota, whereas the periodontal disease microbiota was characterized by a convergent dysbiotic state dominated by pathogenic families, such as *Porphyromonadaceae*. Predicted functionally, this shift was associated with an enrichment of pathways with pro-inflammatory potential, specifically sulfur-containing amino acid metabolism. Additionally, a marked heterogeneity was observed in the composition of healthy microbiota, suggesting alternative stable states, while network analysis revealed a severe reduction in microbial inter-connectivity in periodontal disease. These preliminary findings generate the hypothesis that canine periodontal disease is characterized by “additive dysbiosis” with increased alpha diversity that converges into a uniform, pathogen-enriched structure with collapsed ecological interactions. The loss of potentially protective commensals, such as *Pasteurellaceae*, may act as an ecological trigger for this shift, whereas a healthy oral state may encompass multiple distinct compositional states. These findings provide a novel, hypothesis-generating ecological framework for understanding canine periodontal disease and for developing novel therapeutic strategies.

## Introduction

Paralleling the global rise in companion animal ownership, a socio-cultural phenomenon known as “pet humanization” has emerged, in which pets are increasingly regarded as integral family members^1^. Consequently, the “petconomy,” the industry dedicated to companion animals, has experienced substantial growth^2^. A surging demand for premium products, such as high-quality functional foods, nutritional supplements, and specialized veterinary services, drives this growth. Advances in veterinary medicine have extended the average lifespan of companion animals^3^; however, preventing and managing age-related chronic and degenerative diseases are a critical challenge in veterinary healthcare^4^.

Periodontal disease, a common oral disease in dogs, is one of the most prevalent health problems, affecting dogs across all ages and breeds^5^. Periodontitis is a chronic inflammatory disease of the periodontal tissues, caused by microorganisms within dental plaque. Without treatment, the condition typically begins as mild gingivitis and progresses to destruction of the periodontal ligament, cement, and alveolar bone, ultimately leading to tooth loss^6^. Furthermore, the importance of oral health is highlighted by a “systemic link,” which suggests that periodontal disease is not merely a localized ailment. This link suggests that oral pathogens and inflammatory mediators can translocate from the periodontal tissues into the systemic circulation, thereby contributing to the development or exacerbation of diseases in distant organs, including the heart, kidneys, and liver^7^. In response to these serious health implications, various commercial products, including functional foods, dental chews, and oral care items, have been marketed for periodontal disease prevention and management. However, robust scientific evidence validating their efficacy and elucidating their mechanisms of action, particularly in the context of canine physiology, is lacking^8^.

Recently, the paradigm in biomedical research has shifted from a focus on individual pathogens to the ecological balance of the entire microbial community. In this context, it is crucial to distinguish between the “microbiota,” which refers to the community of microorganisms itself (i.e., “who is there”), and the “microbiome,” which encompasses the microbiota’s collective genetic content and functional potential (i.e., “what they can do”)^9^. In human periodontitis research, the concept of a healthy oral state in symbiosis, where diverse microorganisms interact to maintain stability, is well established. Correspondingly, periodontal disease is considered a state of dysbiosis, in which an ecological imbalance is marked by the collapse of this stability, pathobiont overgrowth, and the restructuring of the entire community^10^. The canine oral cavity also harbors a complex microbiota. Similar to humans, alterations in the canine microbial ecosystem play a key role in the onset and progression of periodontal disease^11^. However, the canine oral microbiota remains poorly understood, as distinct factors, such as diet, oral pH, and genetic background, influence it. A comprehensive picture is yet to be drawn regarding the specific differences between healthy and periodontal disease-associated communities, the identity of keystone taxa driving the disease, and the functional consequences of these community shifts^11^.

Therefore, this exploratory pilot study aimed to address this knowledge gap by employing next-generation sequencing (NGS) technology to comprehensively compare and analyze the oral microbiota of clinically healthy dogs and those with periodontal disease. We hypothesized that the oral microbiota in periodontal disease-afflicted dogs would present a distinct and consistent “dysbiotic signature” compared to that of healthy dogs, characterized by alterations in community structure, interaction networks, and predicted functional potential. This study provides preliminary insights and generates new hypotheses regarding the microbial etiology of canine periodontal disease, ultimately laying the groundwork for the development of future evidence-based diagnostic and therapeutic strategies.

## Results

### Structural divergence and increased alpha diversity of the periodontal disease-associated microbiota

**Fig. 1:**
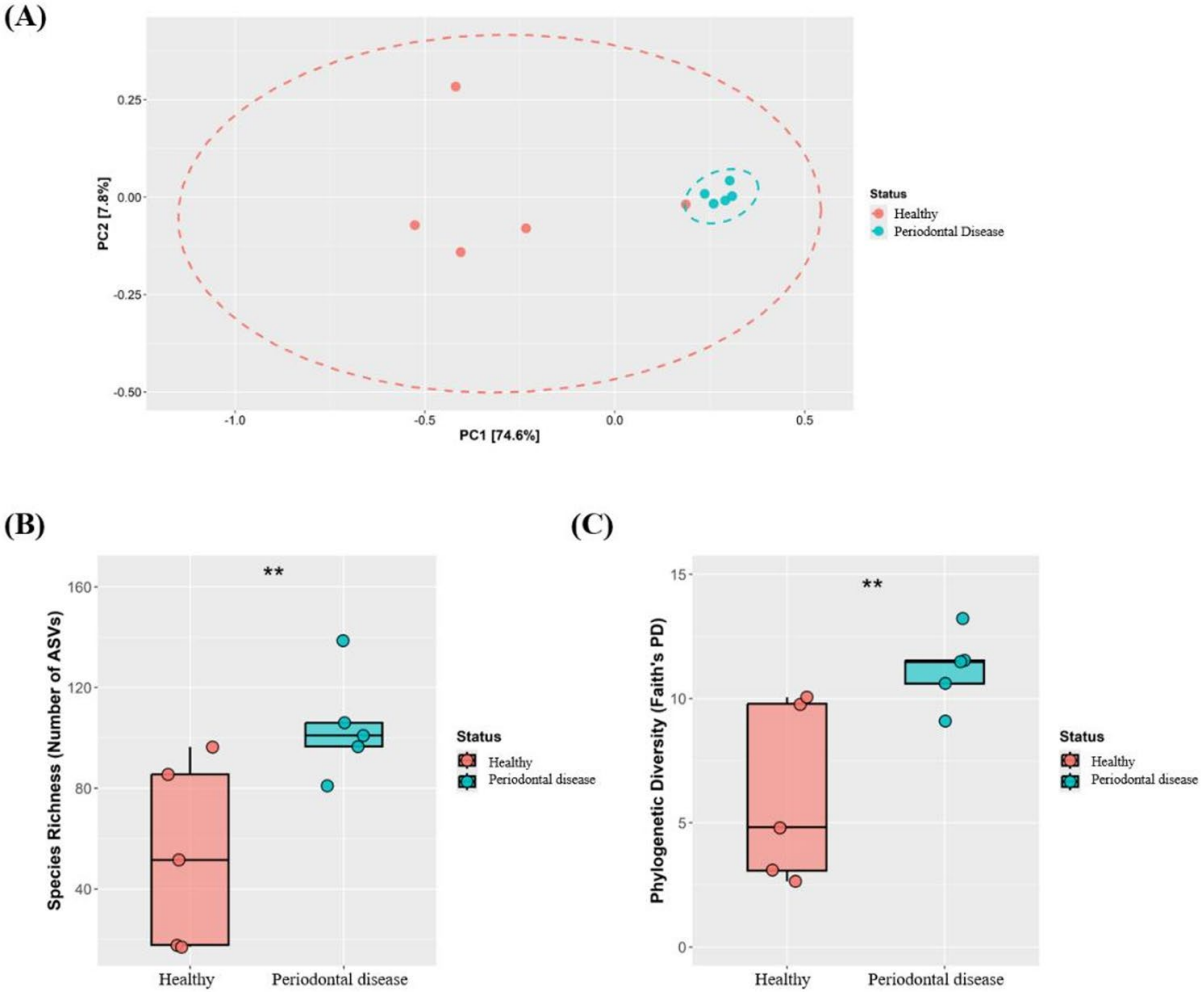
Comparison of oral microbiota between Healthy and periodontal disease canines. (**A**) Principal Coordinate Analysis (PCoA) of weighted UniFrac distances. Statistical significance of the separation was confirmed by age-adjusted marginal PERMANOVA (*R*^*2*^ = 0.385, *p* = 0.009). (**B**) Comparison of species richness (observed amplicon sequence variants [ASVs]) between groups. (**C**) Comparison of phylogenetic diversity (Faith’s PD) between the groups. The significance levels are denoted as follows: * *p* < 0.1, ** *p* < 0.05, *** *p* < 0.01, **** *p* < 0.001.

To compare the overall structure of the oral microbial communities, beta diversity was first evaluated. Principal coordinate analysis (PCoA) based on weighted UniFrac distances revealed a clear separation trend between the healthy and periodontal disease groups (Fig. 1A). The periodontal disease group formed a tight, homogenous cluster, indicating low intra-group variation, whereas the healthy group was more broadly dispersed, suggesting high inter-individual variation. To account for the significant age difference between the groups (Table 1), we performed a permutational multivariate analysis of variance (PERMANOVA) with age as a covariate. The results indicated that even after adjusting for age, the clinical status (‘Group’) remained a highly significant factor explaining the community variance (*R*^2^ = 0.385, *p* = 0.009). This suggests that the observed dysbiosis was strongly associated with the disease state itself, independent of age. A key feature of this dysbiosis appeared to be a paradoxical and significant increase in alpha diversity. Specifically, the median species richness in the periodontal disease group was 102 observed Amplicon sequence variants (ASVs) (interquartile range [IQR], 99–105), significantly higher than the median of 52 ASVs (IQR, 20–88) in the healthy group (Wilcoxon rank-sum test, *p* < 0.05; Fig. 1B). Similarly, phylogenetic diversity (PD) was also significantly elevated in the periodontal disease group (median Faith’s PD, 11.4; IQR, 10.9–11.8) compared to that in the healthy group (median Faith’s PD, 4.8; IQR, 3.2–9.8; *p* < 0.05; Fig. 1C).

**Table 1:** Quantitative comparison of topological properties between Healthy and Periodontal disease microbial networks.

Network Metric	Healthy (H)	Periodontitis (P)
Number of Nodes	58	52
Number of Edges	646	381
Average Degree	22.28	14.65
Edge Density	0.391	0.287
Modularity	0.440	0.364
Transitivity	0.867	0.564

All metrics were calculated using the igraph package in R, based on Spearman’s rank correlation coefficients with a unified threshold of ∣ρ∣ ≥ 0.60 and *p* < 0.05.

### Key taxonomic shifts differentiating healthy and periodontal disease associated oral microbiomes

**Fig. 2:**
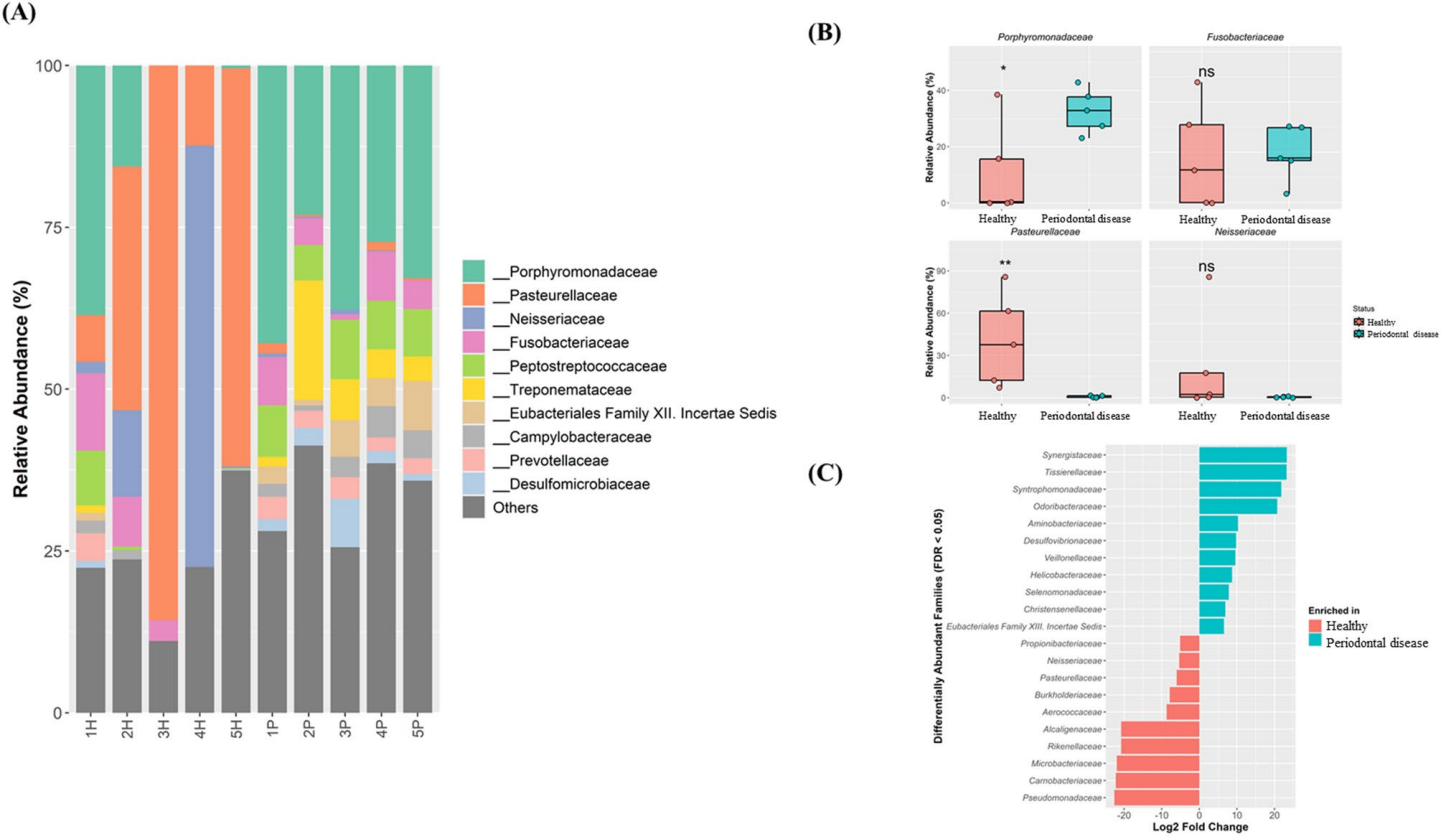
Taxonomic differences in oral microbiota at the family level. (**A**) Relative abundance of the top 10 bacterial families and “Others” in each sample. (**B**) Comparison of the relative abundances of four selected families between the healthy and periodontal disease groups. (**C**) Differentially abundant families identified by DESeq2 (false discovery rate [FDR] < 0.05) were plotted as log2 fold change. Asterisks in (**B**) indicate significance levels: * *p* < 0.1, ** *p* < 0.05, *** *p* < 0.01, **** *p* < 0.001.

Taxonomic composition was analyzed at the family level to identify the specific taxa driving community separation. Analysis of the relative abundance profiles indicated compositional patterns that differed markedly between the two clinical states (Fig. 2A). Specifically, the microbiota of healthy dogs were generally characterized by a high abundance of *Pasteurellaceae*. However, a notable exception was observed in one individual (4H), which was dominated by *Neisseriaceae*, indicating a degree of inter-individual heterogeneity. In contrast, the microbial communities in dogs with periodontal disease exhibited a remarkably consistent and homogeneous structure, which was consistently and overwhelmingly dominated by the family *Porphyromonadaceae*. Four representative families were selected for direct comparison to investigate the key patterns observed in the abundance profiles, including dominant families, inter-group contrasts, and unique individual variations (Fig. 2B). *Pasteurellaceae*, a key family in the healthy group, was significantly depleted in the periodontal disease group (*p* < 0.01). Conversely, *Porphyromonadaceae*, which provides a visual hallmark of the disease state, was markedly higher in the periodontal disease group, demonstrating marginal statistical significance (*p* < 0.1) alongside high variability within the healthy group. In contrast to these consistent group-level shifts, the other selected families were characterized by high inter-individual variability, which precluded a significant difference. For instance, the abundance of *Fusobacteriaceae* was consistently elevated in the periodontal disease group but exhibited high variability among healthy individuals. Conversely, *Neisseriaceae* abundance was uniformly low in all dogs with periodontal disease but varied widely within the healthy group, driven by an exceptionally high abundance in individual 4H. A differential abundance analysis was performed using DESeq2 to identify an objective and statistically stringent list of biomarkers (FDR < 0.05; Fig. 2C). This analysis had two key purposes. First, it supported several of the initial visual observations, thereby identifying *Pasteurellaceae* as a robust biomarker of a healthy state. Second, it highlighted several novel biomarkers not readily apparent in the abundance plots by identifying less-abundant families that were consistently associated with the periodontal disease state. Despite its ecological dominance in the disease group, *Porphyromonadaceae* was not identified as a significant biomarker using this method, suggesting a discrepancy between visual dominance and consistent statistical differentiation.

### Predicted shift in the metabolic potential of the dysbiotic oral microbiota

**Fig. 3:**
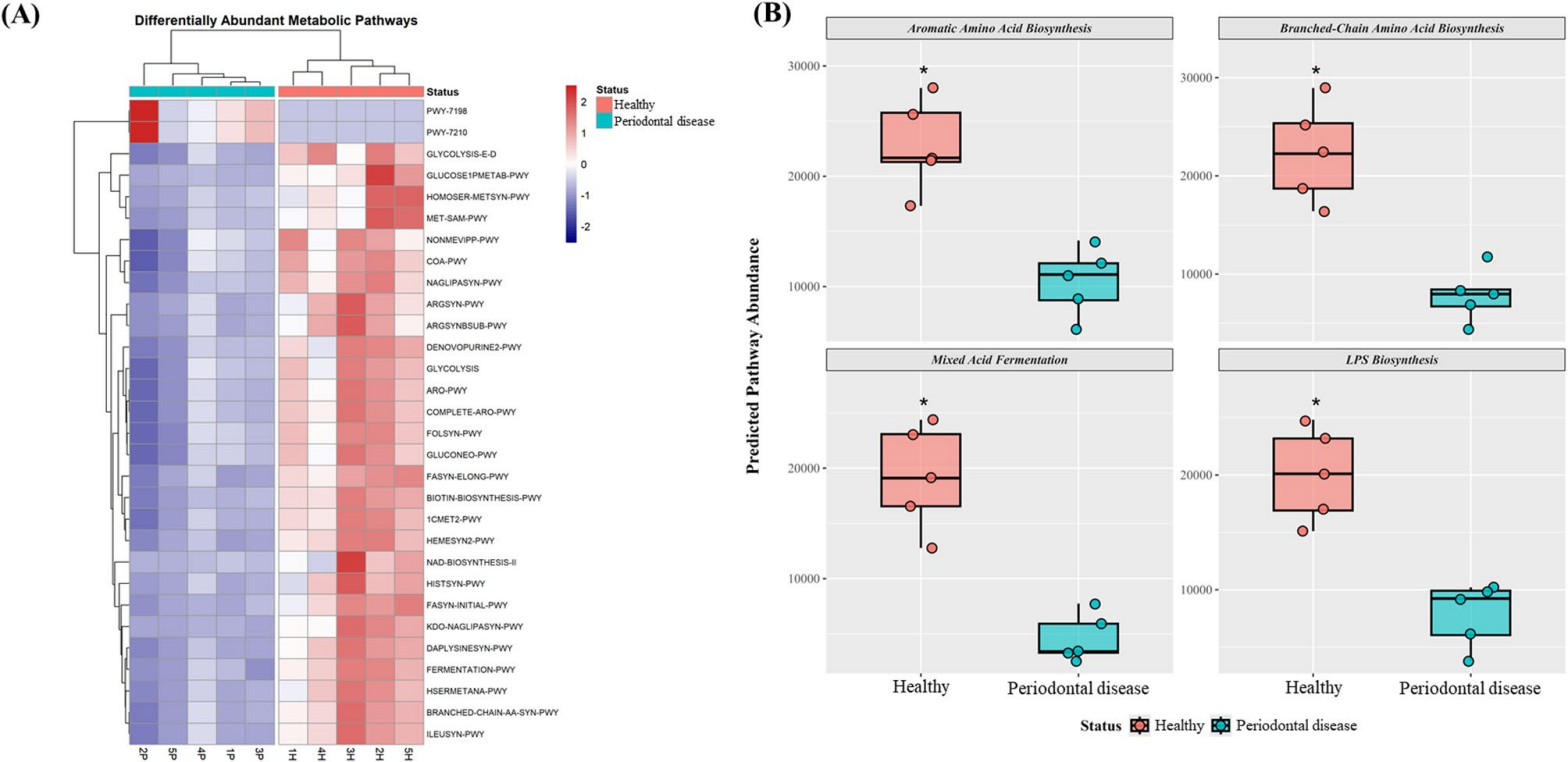
Predicted metabolic potential of oral microbiota in Healthy and Periodontal disease canines. (**A**) Heatmap of the top 30 differentially abundant metabolic pathways (FDR < 0.05). Each row represents a predicted pathway, scaled by Z-score. (**B**) Boxplots comparing the predicted abundance of four selected pathways representing biosynthesis potential (Aromatic and Branched-Chain Amino Acid Biosynthesis) and pathogenicity-associated sulfur metabolism (Methionine Biosynthesis, S-adenosyl-L-methionine [SAM] Cycle) between the Healthy (*n* = 5) and Periodontal disease (*n* = 5) groups. Asterisks in (**B**) indicate unadjusted significance levels: * *p* < 0.1, ** *p* < 0.05.

The metabolic potential of the oral microbiomes was predicted using Phylogenetic Investigation of Communities by Reconstruction of Unobserved States 2 (PICRUSt2) to investigate the functional consequences of the observed taxonomic shifts. The overall accuracy of the predictions was adequate, as indicated by a low mean nearest sequenced taxon index score across all samples (mean ± standard deviation: 0.16 ± 0.08). To rigorously address the risk of false positives inherent in testing numerous pathways, p-values from the Wilcoxon rank-sum test were adjusted using the Benjamini–Hochberg False Discovery Rate (FDR) method. Remarkably, despite the small sample size of this pilot study, numerous pathways remained highly significant even after this stringent correction (FDR < 0.05). We visualized the top 30 pathways ranked by their lowest FDR values in a heatmap, which indicated a clear separation of the functional profiles between the two groups (Fig. 3A).

Hierarchical clustering of the samples based on the abundances of these pathways distinctly segregated the healthy and periodontal disease samples into two separate clusters, thereby corroborating the beta diversity analysis of the community structure. Clustering revealed two major functional modules. The first module, which was predominantly upregulated in the healthy group, was largely composed of biosynthetic pathways essential for community maintenance and growth. In contrast, the second module, which was consistently upregulated in the periodontal disease group, included several pathways associated with anaerobic metabolism and the production of pro-inflammatory bacterial components. The abundances of the four representative pathways were compared to further examine these key functional differences (Fig. 3B). The pathways for the biosynthesis of aromatic amino acids (ARO-PWY) and branched-chain amino acids (BRANCHED-CHAIN-AA-SYN-PWY), which are essential building blocks for protein synthesis, were significantly more abundant in the healthy group than in the periodontal disease group (FDR < 0.05). Conversely, pathways related to mixed acid fermentation (FERMENTATION-PWY), a process that produces tissue-damaging acidic byproducts, and lipopolysaccharide (LPS) biosynthesis (NAGLIPASYN-PWY), a key endotoxin that triggers host inflammatory responses, were significantly enriched in the periodontal disease group (FDR < 0.05).

### Structural disintegration of microbial co-occurrence networks in the periodontal disease -associated ecosystem

**Fig. 4:**
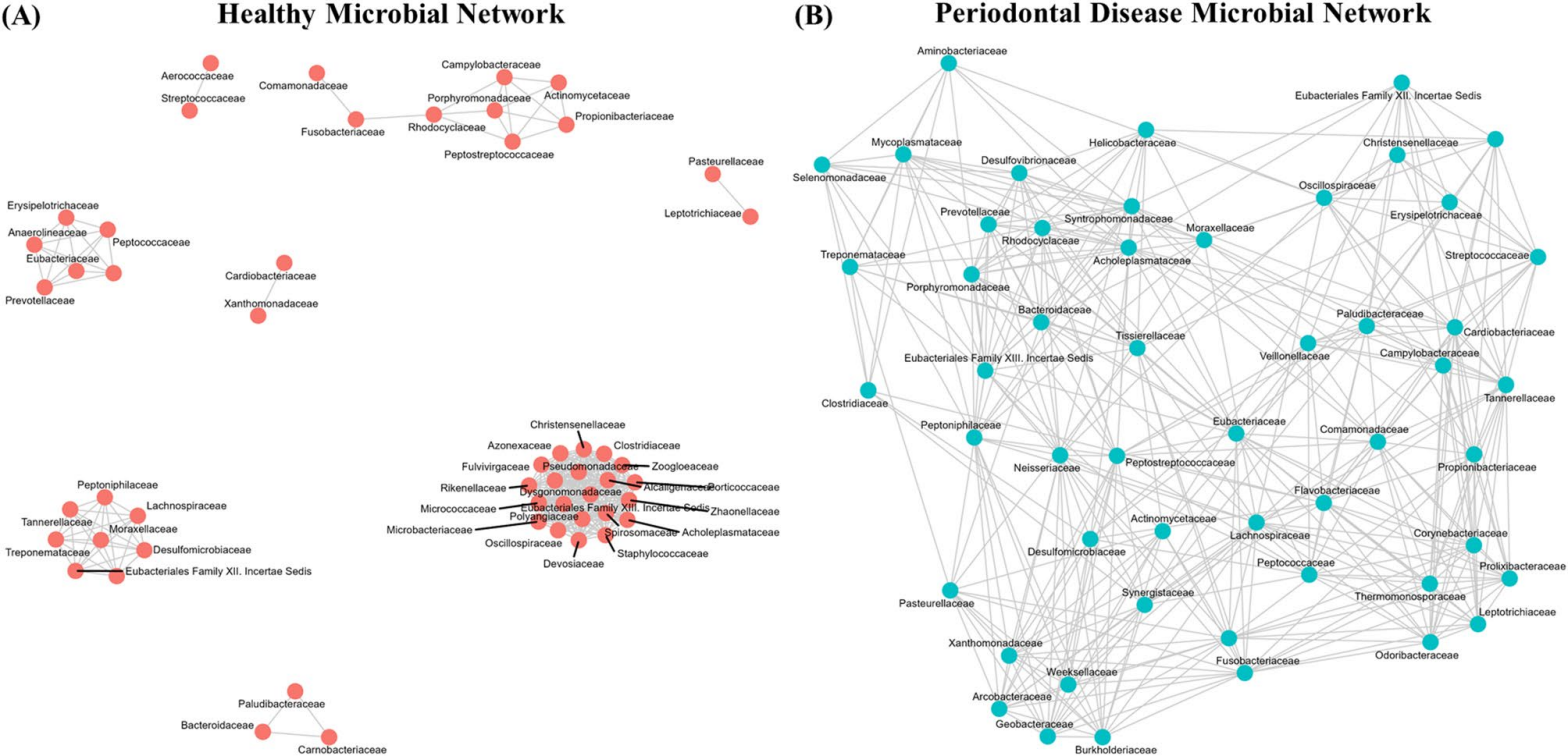
Comparison of microbial co-occurrence networks at the family level. Networks were constructed for the (**A**) Healthy and (**B**) periodontal disease groups based on Spearman’s rank correlations (*ρ*). A unified threshold of |ρ|≥ 0.60 (*p* < 0.05) was applied to both groups to ensure a balanced structural comparison. Nodes represent bacterial families (colored by clinical status), and edges indicate significant correlations.

Co-occurrence networks were constructed separately for the healthy and periodontal disease groups using Spearman’s rank correlation analysis to explore in inter-taxonomic relationships at the family level. To ensure a valid structural comparison between the two ecological states, a unified threshold (|ρ|≥ 0.60, *p* < 0.05) was applied to both groups. Distinct network topologies were observed between the two states (Fig. 4). The healthy oral microbiota formed a highly interconnected and cohesive network, characterized by large, densely populated clusters where numerous bacterial families maintained strong associations. This architecture suggests a resilient community structure underpinned by extensive cooperative interactions. In contrast, the periodontal disease-associated network exhibited signs of structural disintegration and a loss of modular organization. The topology appeared more fragmented, with fewer central hubs connecting the community elements. This visual observation was strongly supported by quantitative topological analysis (Table 2). The healthy network displayed substantially higher connectivity and complexity compared to the periodontal disease network, as evidenced by a significantly greater number of edges (646 vs. 381), higher average degree (22.28 vs. 14.65), and markedly higher transitivity (0.867 vs. 0.564). Furthermore, the identity of the central hubs within these networks shifted drastically. In the robust healthy network, commensal taxa such as *Bacteroidaceae* and *Christensenellaceae* acted as highly connected hubs. In contrast, the fragmented periodontal disease network was primarily driven by established periodontal pathogens or pathobionts, including *Campylobacteraceae*, *Prevotellaceae*, and *Tannerellaceae*. Collectively, this quantitative decline in topological metrics and the shift in keystone taxa indicate a profound loss of ecosystem cohesion during the progression of canine periodontal disease.

**Table 2:** Demographic and clinical characteristics of dogs included in the study.

Sample number	Sex	Age (years)	Race	Weight (kg)	Diet type	Clinical status	Dental calculus	Halitosis	Tooth exfoliation	B.S
*Healthy periodontium (H)*										
1H	M	1.5	Bichon Frise	4.9	CD	–	–	–	–	3
2H	M	10	Maltese	3.8	CD	–	○	–	–	3
3H	F	8	Pomeranian	3.76	CD	–	○	–	–	4
4H	F	1.8	Pomeranian	2.7	CD	–	–	–	–	3
5H	F	8 mon	Poodle	2.9	CD	–	–	–	–	3
*Periodontal disease (P)*										
1P	F	11.6	Yorkshire Terrier	3.1	CD	○	○	○	–	5
2P	M	13.6	Pomeranian	1.8	CD/HM	○	○	○	○	2
3P	F	7	Maltese	3.8	CD	○	○	○	○	3
4P	F	8	Maltese	3.1	CD/HM	○	○	○	–	3
5P	F	11	Maltese	3.1	CD/HM	○	○	○	○	4

M, male; F, female; mon, months; CD, commercial diet; HM, homemade diet. B.S, Body Condition Score (on a 9-point scale).

Symbols: ○, present; -, absent.

### Heterogeneity in healthy oral microbiota and convergence toward a dysbiotic state in periodontal disease

**Fig. 5:**
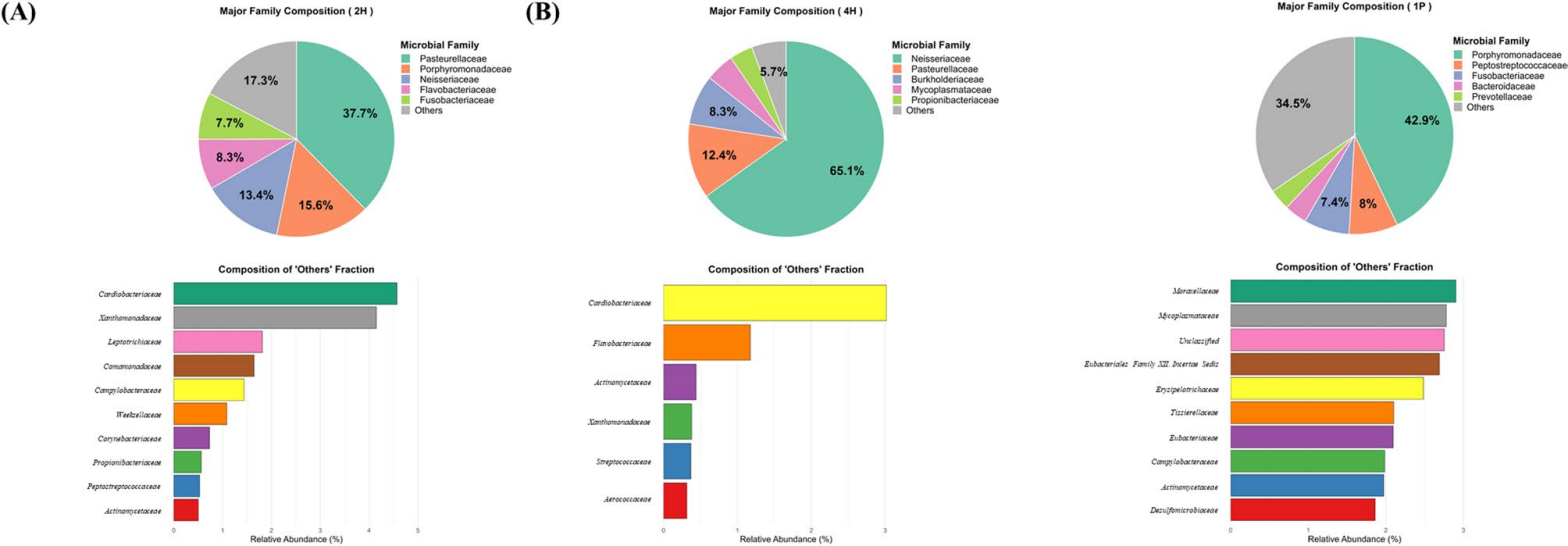
Comparative analysis of individual microbiota profiles in healthy and periodontal disease canines. Composition of major bacterial families for three representative samples: (**A**) Healthy sample dominated by *Pasteurellaceae* (2H), (**B**) a healthy sample dominated by *Neisseriaceae* (4H), and (**C**) a representative periodontal disease sample dominated by *Porphyromonadaceae* (1P). The number of bars shown in the “Others” plots (bottom) reflects the actual number of specific families detected with a relative abundance > 0% in each individual sample (e.g., 6 bars for Sample 4H and 10 bars for Samples 2H/1P).

The family-level profiles of three distinct phenotypes were examined in detail to highlight the contrasting patterns of compositional variability between the groups, namely the heterogeneity in health and the homogeneity in periodontal disease (Fig. 5). Healthy microbiota were characterized by a marked compositional heterogeneity. For example, the profile of one healthy dog (2H) was co-dominated by *Pasteurellaceae* (37.7%) and *Porphyromonadaceae* (15.6%) (Fig. 5A). In contrast, another healthy dog (4H) exhibited a profile overwhelmingly dominated by *Neisseriaceae* (65.1%), which was found in minimal abundance in dog 2H (< 1%) (Fig. 5B). This observation indicated that a healthy oral microbiota can be characterized by multiple distinct compositional states. Conversely, the profile of sample 1P, from a dog with periodontal disease, exhibited a convergent profile dominated by *Porphyromonadaceae* (42.9%), with significant contributions from *Peptostreptococcaceae* (8.0%) and *Fusobacteriaceae* (7.4%) (Fig. 5C). These findings support the hypothesis that a healthy oral microbiota can exist in multiple distinct compositional states; however, the dysbiotic state in periodontal disease converges to a more uniform disease-associated structure.

## Discussion

This exploratory pilot study provides a comprehensive multi-angle characterization of the oral microbiota of healthy canines and those with periodontal disease. Moreover, the study presents several key findings that challenge the existing understanding of canine periodontal dysbiosis. The central finding was that canine periodontal disease appears to be characterized by additive dysbiosis, a concept we frame as a hypothesis-generating model for this disease state^12^. Consistent with the additive dysbiosis model, the periodontal disease group exhibited a significant and paradoxical increase in alpha diversity (Fig. 1). This finding contrasts with the diversity loss consistently reported as a hallmark of dysbiosis in various conditions, including obesity, metabolic disorders, and inflammatory bowel disease (IBD)^13,14^. This result strongly suggests that canine periodontal disease is not merely a process of loss of healthy bacteria. This condition is a more complex and active process wherein the chronic inflammatory environment creates new ecological niches for the invasion and establishment of diverse opportunistic pathogens, a mechanism that has been increasingly recognized in host–microbe interactions^15^.

A key constituent of the healthy oral ecosystem consistently identified in this study was the family *Pasteurellaceae*. This family was one of the dominant taxa within the healthy group (Fig. 2A) and the most significant biomarker for the healthy state by DESeq2 analysis (Fig. 2C). Members of the family *Pasteurellaceae* are typically aerobic or facultatively anaerobic and are key early colonizers of oral mucosal surfaces under normal oxygen tension^16^. Notably, their ecological role is critical in maintaining a microaerophilic environment in the healthy gingival sulcus^17^. A key metabolic trait of this family is its strong nitrate-reducing ability, which leads to the production of nitric oxide (NO) in host salivary glands^18^. This bacterial-derived NO acts as a crucial innate immune mechanism at physiological concentrations, thereby inhibiting anaerobic pathogens via its potent antimicrobial activity^18^. This protective role is distinct from the cytotoxic effects of high NO concentrations produced during inflammation. This finding is consistent with human oral microbiome studies that have also demonstrated a robust positive correlation between the abundance of nitrate-reducing bacteria and oral health^19^. Similar findings have been reported in the oral microbiome of healthy cats, where species belonging to the family *Pasteurellaceae* are highly abundant^20^. Therefore, the substantial loss of *Pasteurellaceae* in the periodontal disease group had several critical implications for the ecological stability of the oral cavity. First, this loss may equate to the functional collapse of the “nitrate–nitrite–nitric oxide pathway,” which is a primary antimicrobial defense system within the oral ecosystem. Second, the loss of these oxygen-consuming bacteria likely facilitated the overgrowth of obligate anaerobes, such as *Porphyromonadaceae*, by creating a more anaerobic oral environment. These possibilities suggest that the disappearance of *Pasteurellaceae* is not merely a consequence of dysbiosis, but rather a decisive ecological trigger that initiates the shift toward a pathogenic community.

In contrast to the healthy group, the periodontal disease group was characterized by an overwhelming visual co-dominance of *Porphyromonadaceae* and *Fusobacteriaceae* (Fig. 2A and B). Species within these two families are well-established as key periodontal pathogens in both humans and dogs^11,21^. In particular, *Porphyromonas* species function as keystone pathogens by secreting potent proteases (e.g., gingipains) that directly destroy host tissues, evade immune responses, and remodel the local microenvironment to favor the proliferation of the entire pathogenic community^10^. Furthermore, *Fusobacterium* species are critical bridging organisms that facilitate the coaggregation of early and late colonizers, thereby physically scaffolding the pathogenic biofilm^22^. Paradoxically, despite its overwhelming visual dominance in the periodontal disease group, DESeq2 analysis did not identify *Porphyromonadaceae* as a significant biomarker (Fig. 2C). This discrepancy suggests that *Porphyromonadaceae* may function not as a universal initiator of the disease, but rather as a pathobiont, which is a common resident of the healthy oral cavity that proliferates opportunistically once the defenses of the ecosystem are compromised. This interpretation is strongly supported by recent large-scale findings, where *Porphyromonas* was identified as one of the most abundant genera in healthy dogs via NGS^23^. The high and variable abundance of *Porphyromonadaceae* in the healthy state likely precluded it from meeting the stringent FDR cutoff for a consistent biomarker. Furthermore, this model is consistent with findings in human periodontitis, where the abundance of *Porphyromonas gingivalis* correlated with disease severity rather than with disease onset^24^. Ultimately, this observation highlights a fundamental discrepancy between ecological dominance and consistent statistical differentiation in microbiome data.

The paradoxical increase in alpha diversity observed in the periodontal disease state (Fig. 1B and C) contrasts with the typical patterns observed in numerous dysbiotic models. This traditional view of dysbiosis is exemplified by IBD, in which barrier dysfunction and widespread inflammation lead to the large-scale extinction of commensal anaerobes, which drastically reduce overall diversity^14^. This phenomenon represents a classic model of subtractive dysbiosis, an ecological state characterized by the depletion of the ecosystem. In contrast, the phenomenon observed in this study is best explained by the unique pathological environment of periodontal disease. Chronic gingival inflammation promotes bleeding and exudation of gingival crevicular fluid, a process that creates a new, nutrient-rich environment abundant in proteins and heme-derived factors^25^. Unlike the habitat destruction characteristic of IBD, this altered oral environment adds new nutritional sources, thereby creating novel ecological niches that facilitate the colonization and growth of fastidious anaerobic bacteria that cannot survive. This model is consistent with findings from human periodontitis studies, where a transient increase in species richness was observed during disease progression owing to the addition of specific pathogenic groups^26^. Thus, the observed high diversity supports the “additive dysbiosis” model, suggesting that the disease is driven by the recruitment of a pathogenic consortium rather than a simple collapse of the healthy community.

To provide a deeper, structural perspective on this dysbiotic shift, we analyzed the microbial co-occurrence networks (Fig. 4). The unified threshold analysis quantitatively demonstrated that the healthy microbiota forms a highly interconnected and robust network, characterized by significantly higher density and transitivity (Table 2). This complex architecture suggests a resilient ecosystem stabilized by numerous cooperative and competitive interactions. In stark contrast, the periodontal disease microbiota exhibited a severe structural disintegration. This was not merely a reduction in connections but a fundamental shift in network topology, from a cohesive, modular structure to a fragmented and loosely connected state. This network collapse provides a powerful mechanistic explanation for the loss of ecological resilience in the diseased state.

A fascinating aspect of our findings is the functional decoupling between ecological dominance (high relative abundance) and network centrality (hub taxa). While families like *Pasteurellaceae* or *Neisseriaceae* dominated the biomass in healthy individuals (Fig. 2A, Fig. 5), the network’s structural integrity was anchored by a different set of highly connected hubs, such as *Bacteroidaceae* and *Christensenellaceae* (Fig. 4). This suggests a division of labor in the healthy ecosystem: dominant taxa may act as environmental engineers by controlling resources like oxygen, while network hubs act as community stabilizers by mediating key inter-species interactions. Conversely, in the fragmented periodontal disease network, the remaining connections were centered around established periodontal pathogens, including *Tannerellaceae* and *Prevotellaceae*. Although not the most abundant visually, these taxa are known members of the pathogenic ‘Red’ and ‘Orange’ complexes and may function as keystone pathogens, orchestrating the dysbiotic community even at lower abundances. This highlights that understanding canine periodontal disease requires viewing the microbiome not just through the lens of ‘who is most abundant,’ but also ‘who connects whom’ within the collapsing ecosystem.

Profound alterations in community structure and interactions were paralleled by shifts in the predicted functional potential of the microbiota (Fig. 3). A healthy community was characterized by a stable, biosynthesis-centric metabolism, such as the biosynthesis of aromatic and branched-chain amino acids. In contrast, the periodontal disease community exhibited a marked enrichment in pathways associated with sulfur-containing amino acid metabolism, specifically methionine biosynthesis and the S-adenosyl-L-methionine (SAM) cycle. This finding has direct clinical implications for the pathophysiology of periodontal disease. LPS is a major component of the gram-negative bacterial cell wall and a potent endotoxin that triggers a strong inflammatory response by activating toll-like receptor 4 on host immune cells^27^. Periodontal pathogens, particularly those within the *Porphyromonadaceae* and *Fusobacteriaceae* families, actively metabolize these sulfur-containing amino acids into volatile sulfur compounds (VSCs), such as hydrogen sulfide and methyl mercaptan^28^. These VSCs are not only the primary culprits behind halitosis—a clinical sign explicitly documented in all of our periodontal disease cohort (Table 1)—but are also highly cytotoxic. They can directly promote gingival inflammation, induce apoptosis in host cells, and accelerate periodontal tissue destruction^29^. Therefore, predicted enrichment of sulfur metabolism in the periodontal disease community provides a strong mechanistic hypothesis linking that this microbial consortium to a hallmark clinical sign of the disease: halitosis and localized tissue damage. This predicted functional shift, based on 16S rRNA gene data, warrants cautious interpretation, as it may not reflect actual gene expression or metabolic activity. However, this prediction is consistent with the observed taxonomic shifts and provides a strong hypothesis for a feedback loop in which the dysbiotic community continually stimulates the host immune system and damages local tissues, thereby exacerbating chronic inflammation.

Although a definitive conclusion cannot be drawn from the small sample size, one of the most intriguing hypothesis-generating observations of this study is the heterogeneity of health. The observation that the healthy group included individuals dominated by *Pasteurellaceae* (2H) and *Neisseriaceae* (4H) (Fig. 5) suggests the possibility that a healthy microbiota may not be a single, defined state. This observation is consistent with the ecological theory of alternative stable states, which explains heterogeneity in human microbiomes^30^. According to this theory, different taxonomic configurations maintain a stable equilibrium by performing similar overall functions through functional redundancy. Thus, core ecological roles necessary for health, such as oxygen consumption and pathogen inhibition, can be fulfilled using different combinations of microorganisms. Thus, the *Pasteurellaceae*- and *Neisseriaceae*-dominant phenotypes observed in this study may represent two such alternative stable states, which are functionally equivalent despite being taxonomically distinct. This finding suggests that a more realistic and effective approach for future probiotic or microbiome-based therapies may foster resilience in one of several possible healthy states, rather than aiming to restore a single, idealized golden standard community.

Finally, this study has several important limitations. This study is best contextualized as an exploratory investigation intended to generate hypotheses rather than to provide definitive conclusions. The sample size (*n* = 5 per group) is small, which may have limited the statistical power to detect less abundant taxa and may have affected the generalizability of the findings. The use of supragingival plaque, while practical for non-invasive sampling, may not fully capture the dynamics within the subgingival pocket, the primary site of inflammation. Additionally, the cross-sectional design and uncontrolled variables such as breed and diet represent potential confounding factors. Although the difference in the average age between the two groups reflects the clinical reality that periodontal disease is predominantly diagnosed in older animals, age itself remains a potential confounding variable^31^. However, our PERMANOVA analysis adjusted for age indicated that disease status was a more significant driver of community structure than age in our cohort. Nevertheless, the distinct and significant differences observed across ecological, taxonomic, and predicted functional analyses are remarkable despite these limitations. This finding suggests the presence of a strong biological signal that transcends the small sample size. Therefore, the findings of this study serve as critical preliminary data and provide a robust foundation for hypotheses that warrant validation in future longitudinal studies involving larger, age-matched, and breed-controlled populations.

## Conclusion

These preliminary findings suggest that canine periodontal disease is characterized by a convergent dysbiosis with increased diversity, accompanied by a shift toward predicted pathogenic metabolic potential and a collapse of the ecosystem network (Figs. 1, 3, and 4). Furthermore, the study findings suggest that a healthy oral microbiota may exist in multiple alternative stable states (Fig. 5). These findings provide a novel, hypothesis-generating ecological framework for understanding of the microbial pathogenesis of canine periodontal disease and lay the groundwork for the development of future evidence-based diagnostic and therapeutic strategies to restore ecological stability.

## Methods

### Ethics statement

Informed consent was obtained from all dog owners prior to their participation in the study. All samples were collected by a licensed veterinarian during routine health examinations or clinically necessary dental procedures at a local veterinary hospital in Jeongeup, Korea. The study protocol was reviewed and approved by the internal review board of the Center for Industrialization of Agricultural and Livestock Microorganisms (CIALM). All methods were carried out in accordance with relevant guidelines and regulations for veterinary care to ensure animal welfare, and the study is reported in compliance with the ARRIVE guidelines. As samples were obtained non-invasively during standard clinical practice for diagnostic purposes, a formal institutional animal care and use committee (IACUC) approval number was not required per the determination of the review board.

### Animal selection and sample collection

Oral swab samples were obtained from a total of 10 client-owned dogs. The demographic and clinical characteristics of the dogs are summarized in Table 1. The healthy group consisted of five dogs (mean age, 4.4 years; breeds: Bichon Frise, Maltese, Pomeranian, Poodle). Although minor dental calculus was noted in two dogs, all dogs in this group were free of clinical signs of gingivitis and periodontal disease. The periodontal disease group included five dogs (mean age, 10.2 years; breeds: Yorkshire Terrier, Pomeranian, Maltese) diagnosed with moderate to severe periodontitis based on veterinary dental assessment, exhibiting clinical signs such as gingivitis, dental calculus, and halitosis. Dogs that had received antibiotic or anti-inflammatory treatments within three months prior to sampling were excluded from the study.

### Sample collection and shipment

Supragingival plaque samples were collected from the buccal surfaces of the maxillary premolars and molars of each dog using sterile swabs. Samples were collected using the DNA/RNA Shield™ SafeCollect™ Swab Collection Kit (Zymo Research, Irvine, CA, USA). After collection, the swabs were immediately immersed in the nucleic acid preservation solution provided in the kit to prevent microbial degradation. The samples were then stored under refrigerated conditions and promptly shipped to Macrogen Inc. (Seoul, Republic of Korea) for analysis.

### 16S rRNA gene sequencing and primary bioinformatic analysis

DNA extraction and sequencing and primary bioinformatics analysis were performed by Macrogen Inc. (Seoul, Republic of Korea). Total genomic DNA was extracted from the samples according to an outsourced protocol, and the near full-length 16S rRNA gene was amplified using the primers 27F and 1492R^11^. The resulting amplicons were sequenced on a PacBio Single Molecule Real-Time sequencing platform to generate high-fidelity long reads.

Sequencing data were processed using the QIIME 2 pipeline^32^. Amplicon sequence variants (ASVs) were generated using the DADA2 plugin^33^, and the taxonomic classification of each ASV was assigned via the Basic Local Alignment Search Tool search^34^ against the National Center for Biotechnology Information 16S database. The final deliverables from this service included a BIOM file (ASV_table.blast_NCBI_16S.biom), an ASV representative sequence FASTA file, and pre-calculated diversity metrics, including a weighted UniFrac distance matrix.

### Predicted functional profile analysis

ASV representative sequence FASTA and BIOM files provided by Macrogen were uploaded to the public Galaxy server to predict the functional potential of the microbial communities^35^. The uploaded files were used as input to execute the Phylogenetic Investigation of Communities by Reconstruction of Unobserved States 2 (PICRUSt2) pipeline^36^. This analysis yielded a table of the predicted MetaCyc metabolic pathway abundances, which were subsequently used for statistical analysis.

### Alpha and beta diversity analysis

Alpha and beta diversities were analyzed based on pre-calculated result files provided by Macrogen. Statistical differences in alpha diversity indices (observed ASVs and Faith’s phylogenetic diversity [PD]^37^) between groups were assessed using the Wilcoxon rank-sum test. For beta diversity, principal coordinate analysis (PCoA) was performed on the weighted UniFrac distance matrix^38^ to visualize the community structure. To assess the influence of age as a potential confounding variable, significant separation of community structures between the healthy and periodontal disease groups was evaluated via a marginal Permutational Multivariate Analysis of Variance (PERMANOVA) (999 permutations) with age included as a covariate using the adonis2 function in the R package vegan^39^.

### Taxonomic and differential abundance analysis

The BIOM file was imported into R using the phyloseq package^40^ to analyze taxonomic compositions. The relative abundance of each sample was calculated at the family level and used for visualization. Differential abundance analysis was performed on the raw count data within the BIOM file using the DESeq2 package to identify microbial biomarkers with significant differences in abundance between the two groups^41^. A Benjamini–Hochberg adjusted p-value (false discovery rate [FDR]) of less than 0.05 was considered statistically significant.

### Co-occurrence network analysis

Co-occurrence networks were constructed for the healthy and periodontal disease groups separately to explore inter-taxonomic relationships at the family level. Spearman’s rank correlation coefficient (ρ) was calculated for all possible pairs of families within each group. To ensure a valid and balanced structural comparison between the two ecological states, a unified threshold of an absolute correlation coefficient |ρ|≥ 0.60 with a significance level of *p* < 0.05 was applied to both networks. Network visualization and analysis (e.g., density, transitivity), and hub identification (degree) were performed using the igraph package^42^.

### Statistical analysis and visualization

For the predicted functional pathway analysis, statistical comparisons between the two groups were conducted using the Wilcoxon rank-sum test. To account for multiple comparisons across all pathways, p-values were subsequently adjusted using the Benjamini–Hochberg False Discovery Rate (FDR) method. The top 30 pathways ranked by the lowest FDR values were selected for heatmap visualization to identify exploratory trends. All data handling, statistical analyses, and figure generation were performed in R (version 4.5.1)^43^ using various packages, including phyloseq (v1.40.0), ggplot2 (v3.4.0)^44^, vegan (v2.6–4)^45^, DESeq2 (v1.36.0), pheatmap (v1.0.12), and igraph (v1.3.5).

## Data Availability

The raw 16S rRNA gene sequencing datasets generated and analysed during the current study are available in the European Nucleotide Archive (ENA) repository, under the BioProject accession number PRJEB106452.
